# Survival and Trends in Annualized Hazard Function by Age at Diagnosis Among Chinese Breast Cancer Patients Aged ≤40 Years: Case Analysis Study

**DOI:** 10.2196/47110

**Published:** 2023-07-18

**Authors:** Jun Wang, Ting Luo, Zhong-zheng Xiang, Ming-min He, Yuan-yuan Zeng, Tian Yang, Xiao-yuan Wei, Siting Yu, Ze-lei Dai, Ning-yue Xu, Chen-feng Tan, Lei Liu

**Affiliations:** 1 Department of Head and Neck Oncology West China Hospital Sichuan University Chengdu China; 2 Breast Disease Center, Cancer Center West China Hospital Sichuan University Chengdu China

**Keywords:** breast cancer, young age, age strata, survival, annual hazard function, China

## Abstract

**Background:**

Young breast cancer patients are more likely to develop aggressive tumor characteristics and a worse prognosis than older women, and different races and ethnicities have distinct epidemiologies and prognoses. However, few studies have evaluated the clinical biological features and relapse patterns in different age strata of young women in Asia.

**Objective:**

We aimed to explore survival differences and the hazard function in young Chinese patients with breast cancer (BC) by age.

**Methods:**

The patients were enrolled from West China Hospital, Sichuan University. The chi-squared test, a Kaplan-Meier analysis, a log-rank test, a Cox multivariate hazards regression model, and a hazard function were applied for data analysis. Locoregional recurrence–free survival (LRFS), distant metastasis–free survival (DMFS), breast cancer–specific survival (BCSS), and overall survival (OS) were defined as end points.

**Results:**

We included 1928 young BC patients diagnosed between 2008 and 2019. Patients aged 18 to 25, 26 to 30, 31 to 35, and 36 to 40 years accounted for 2.7% (n=53), 11.8% (n=228), 27.7% (n=535), and 57.7% (n=1112) of the patients, respectively. The diagnosis of young BC significantly increased from 2008 to 2019. Five-year LRFS, DMFS, BCSS, and OS for the entire population were 98.3%, 93.4%, 94.4%, and 94%, respectively. Patients aged 18 to 25 years had significantly poorer 5-year LRFS (*P*<.001), 5-year DMFS (*P*<.001), 5-year BCSS (*P*=.04), and 5-year OS (*P*=.04) than those aged 31 to 35, 26 to 30, and 36 to 40 years. The hazard curves for recurrence and metastasis for the whole cohort continuously increased over the years, while the BC mortality risk peaked at 2 to 3 years and then slowly decreased. When stratified by age, the annualized hazard function for recurrence, metastasis, and BC mortality in different age strata showed significantly different trends, especially for BC mortality.

**Conclusions:**

The annual diagnosis of young BC seemed to increase in Chinese patients, and the distinct age strata of young BC patients did not differ in survival outcome or failure pattern. Our results might provide strategies for personalized management of young BC.

## Introduction

Breast cancer (BC) is the most frequently diagnosed cancer (approximately 31% of all cancer sites) and is the second cause of mortality among female patients based on a 2022 prediction [1]. Young BC, defined as BC occurring in people aged ≤40 years, has always been a hotly discussed issue due to its lower incidence but poorer prognosis than BC in older patients [2]. The annual number of diagnoses of young BC is approximately 14,000 in the United States, with an estimated incidence of 5% to 7%, while a higher proportion is reported in Asia, up to 20% [3-5]. The diagnosis of young BC has sharply increased in several countries over the last years [3,6].

Young BC patients are more likely to develop aggressive tumor characteristics and have a worse prognosis compared with older patients [7-15]. Previous studies that used population-based data have reported that young BC patients have higher rates of advanced, poorly differentiated tumors, estrogen receptor (ER) negativity, progesterone receptor (PR) negativity, human epidermal growth factor receptor 2 (HER-2) positivity, a higher Ki-67 index, and lymphovascular invasion [7-12]. Young BC patients have also been definitively demonstrated to have a higher proportion of invasive molecular subtypes, including triple-negative and HER-2–positive subtypes; higher rates of distant disease at initial diagnosis; and poor long-term survival outcomes compared with older patients [13-15].

Several studies have reported that the epidemiology and prognosis of young BC also vary in different races and ethnicities [16-18]. The incidence of young BC in the United States is significantly lower than that in Asian countries (approximately 7% vs approximately 20%) [3-6]. Young African-American BC patients have increased risk of BC at a younger age, higher pathological grade, and higher rates of hormone receptor (HR) negativity compared to young White women [16,17]. In addition, young Asian patients have higher proportions of advanced-stage cancers and lower rates of poorly differentiated cancers and invasive BC subtypes (triple-negative and luminal B subtypes) but a better prognosis than young White patients [18]. However, few studies have evaluated the clinical biological features and relapse patterns in different age strata among young women in Asia. Therefore, our study aims to explore the clinicopathological characteristics, survival outcomes, and hazard function of Chinese patients aged ≤40 years by age group (18-25, 26-30, 31-35, and 36-40 years).

## Materials

### Patients

**Figure 1:**
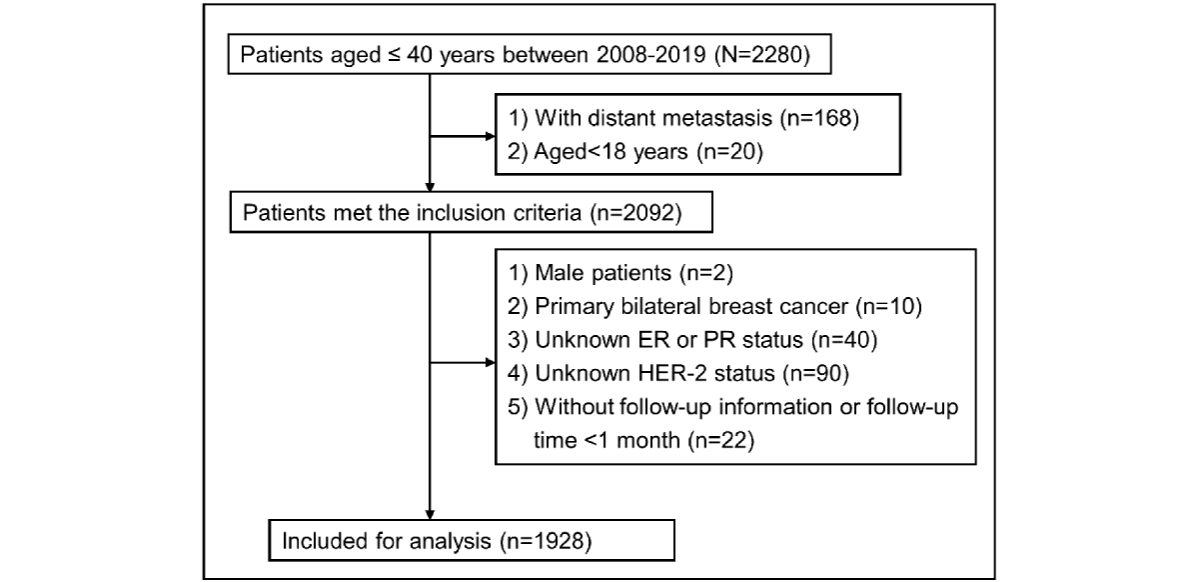
Inclusion flow sheet of patients. ER: estrogen receptor; HER-2: human epidermal growth factor receptor 2; PR: progesterone receptor.

The patient data were extracted from the database of West China Hospital, Sichuan University. The database, the Breast Cancer Information Management System (BCIMS), prospectively collects patient information from medical records on demographics, tumor characteristics, treatment, and follow-up. We included patients based on the following criteria: (1) diagnosis with BC between 2008 and 2019; (2) age ≤40 years; (3) nonmetastatic disease; and (4) availability of detailed information on age, tumor stage, nodal stage, clinical stage, ER status, PR status, HER-2 status, molecular subtype, surgery, neoadjuvant/adjuvant chemotherapy, radiotherapy, endocrine therapy, anti–HER-2 targeted therapy, and follow-up. We excluded patients with the following characteristics: (1) male, 2) age ≤18 years, and (3) bilateral BC. The inclusion flow sheet of the patients is shown in Figure 1.

### Variables

The following patient demographic and clinicopathological variables were included: age (18-25, 26-30, 31-35, and 36-40 years), tumor stage (tumor in situ [TIS], T1, T2, T3, T4), nodal stage (N0, N1, N2, N3), clinical stage (0, I, II, III), pathological grade (well differentiated, moderately differentiated, poorly differentiated/undifferentiated, unknown), ER status, PR status, HER-2 status, molecular subtype (luminal A, luminal B, HER-2 positive, triple-negative), surgery (breast-conserving, mastectomy, unknown), neoadjuvant chemotherapy, adjuvant chemotherapy, radiotherapy, endocrine therapy, and targeted therapy.

### Treatment, Follow-Up, and End Points

Surgery, chemotherapy, and radiotherapy regimens were formulated by a cooperative, multidisciplinary group including surgeons, oncologists, radiotherapy physicians, and patients. Neoadjuvant/adjuvant chemotherapy depended on advanced tumor stage, advanced nodal stage, invasive molecular subtype, such as triple-negative BC, and the willingness to undergo breast-conserving surgery. Radiotherapy was administered to patients receiving breast-conserving surgery with a positive margin and positive axillary lymph nodes. Patients who were HR positive received neoadjuvant or adjuvant endocrine therapy, and patients with HER-2 overexpression were treated with an anti–HER-2 targeted therapy, permitting economic conditions. Follow-up information was collected from medical records, office visits, and telephone calls every 3 months in the first 2 years, semiannually during years 2 to 5 years, and once a year after 5 years. The end points in this study were locoregional recurrence–free survival (LRFS), distant metastasis–free survival (DMFS), breast cancer–specific survival (BCSS), and overall survival (OS). The definitions of LRFS, DMFS, BCSS, and OS were stated in our previous study [19].

### Statistical Analysis

The chi-squared test was used to compare differences in patient baseline characteristics in different age groups. Excel (2016 version; Microsoft Corp) was used to draw variation trends between 2008 and 2019 in the 4 age groups. A Kaplan-Meier analysis was applied to draw survival curves for LRFS, DMFS, OS, and BCSS. A log-rank test was used to compare differences between the 4 groups. A Cox multivariate hazards regression model was used to identify protective and risk factors for predicting LRFS, DMFS, OS, and BCSS. Annualized hazard rates for the whole group and different age strata, defined as percentage of events occurring within a time interval, were calculated using maximum likelihood estimate of a piece-wise exponential model. SPSS (version 25.0; IBM Corp) and Excel were used to analyze and map data. *P* values less than .05 (2-tailed) were considered statistically significant.

### Ethical Considerations

Our study was approved by the Biomedical Ethics Committee of West China Hospital, Sichuan University (2020427). Informed consent was obtained from the participants when they first received treatment in our institution. Patient privacy was well protected due to the deidentification of their information.

## Results

### Patient Characteristics and Treatment Information

A total of 1928 patients met the inclusion criteria and were included for analysis. Patients aged 18 to 25 years, 26 to 30 years, 31 to 35 years, and 36 to 40 years accounted for 2.7% (n=53), 11.8% (n=228), 27.7% (n=535), and 57.7% (n=1112) of the participants, respectively. The proportions of patients at the TIS, T1, T2, T3, and T4 stages were 1.5% (n=28), 36.2% (n=697), 50.5% (n=976), 6.3% (n=121), and 5.5% (n=106), respectively. In addition, 44.6% (n=860), 32.2% (n=621), 11.8% (n=228), and 11.4% (n=219) of the patients were at the N0, N1, N2, and N3 stages, respectively.

The majority of the patients were at an early clinical stage (n=1405, 72.9%), had moderately or poorly differentiated tumors (n=1342, 69.7%), were ER positive (n=1345, 69.8%), were PR positive (n=1288, 66.8%), and were HER-2 negative (n=1383, 71.7%). Regarding molecular subtype, 17.9% (n=346), 54.9% (n=1059), 10.1% (n=194), and 17.1% (n=329) of the patients had the luminal A, luminal B, HER-2–positive, and triple-negative subtypes, respectively. Detailed patient characteristics are presented in Table 1.

In total, 17.4% (n=335) and 79.5% (n=1534) of the patients received breast-conserving surgery and mastectomy, respectively, while 3.1% (n=59) were not treated with surgery or had no record of surgery. In total, 19.8% (n=382) of the patients received neoadjuvant chemotherapy, 89.8% (n=1731) of the patients received adjuvant chemotherapy, and 45.7% (n=881) of the patients were treated with radiotherapy. In addition, 71% (n=1389) of the patients received endocrine therapy and 17.6% (n=340) were treated with targeted therapy (Table 1).

There were no statistical differences in baseline characteristics between the 4 groups (ages 18-25, 26-30, 31-35, and 36-40 years), including tumor stage (*P*=.53), nodal stage (*P*=.65), clinical stage (*P*=.33), ER status (*P*=.42), PR status (*P*=.16), HER-2 status (*P*=.89), pathological grade (*P*=.07), and molecular subtype (*P*=.43). However, patients aged 18 to 25 years were more likely to receive breast-conserving surgery (*P*<.001), adjuvant chemotherapy (*P*=.006), and radiotherapy (*P*=.02), while patients aged 36 to 40 years were more likely to receive mastectomy (*P*<.001). Patients aged 26 to 30 years were more likely to be treated with neoadjuvant chemotherapy (*P*<.001) and targeted therapy (*P*=.04; Table 1).

**Table 1:** Baseline characteristics of overall population.

Variables			Participants (n=1928), n (%)		Participants by age group (years), n (%)									*P* value	
					18-25 (n=53)		26-30 (n=228)		31-35 (n=535)		36-40 (n=1112)				
**Tumor stage**															.53
	Tumor in situ	28 (1.5)		0 (0)		4 (1.8)		10 (1.9)		14 (1.3)					
	T1	697 (36.2)		24 (45.3)		81 (35.5)		187 (35)		405 (36.4)					
	T2	976 (50.5)		22 (41.5)		110 (48.3)		265 (49.4)		579 (52.1)					
	T3	121 (6.3)		4 (7.5)		19 (8.3)		40 (7.5)		58 (5.2)					
	T4	106 (5.5)		3 (5.7)		14 (6.1)		33 (6.2)		56 (5)					
**Nodal stage**															.65
	N0	860 (44.6)		20 (37.7)		101 (44.3)		237 (44.3)		502 (45.1)					
	N1	621 (32.2)		15 (28.3)		71 (31.1)		174 (32.5)		361 (32.5)					
	N2	228 (11.8)		9 (17)		23 (10.1)		65 (12.1)		131 (11.8)					
	N3	219 (11.4)		9 (17)		33 (14.5)		59 (11)		118 (10.6)					
**Clinical stage**															.33
	0	23 (1.2)		0 (0)		4 (1.8)		6 (1.1)		13 (1.2)					
	I	445 (23.1)		17 (32.1)		52 (22.8)		119 (22.2)		257 (23.1)					
	II	937 (48.6)		17 (32.1)		103 (45.2)		262 (49)		555 (49.9)					
	III	523 (27.1)		19 (35.8)		69 (30.2)		148 (27.7)		287 (25.8)					
**Pathological grade**															.07
	Well differentiated	34 (1.8)		0 (0)		7 (3.1)		8 (1.5)		19 (1.7)					
	Moderately differentiated	556 (28.8)		11 (20.8)		60 (26.3)		178 (33.3)		307 (27.6)					
	Poorly differentiated/undifferentiated	786 (40.8)		21 (39.6)		88 (38.6)		201 (37.6)		476 (42.8)					
	Unknown	552 (28.6)		21 (39.6)		73 (32)		148 (27.6)		310 (27.9)					
**Estrogen receptor status**															.42
	Positive	1345 (69.8)		33 (62.3)		162 (71.1)		364 (68)		786 (70.7)					
	Negative	583 (30.2)		20 (37.7)		66 (28.9)		171 (32)		326 (29.3)					
**Progesterone receptor status**															.16
	Positive	1288 (66.8)		30 (56.6)		147 (64.5)		349 (65.2)		762 (68.5)					
	Negative	640 (33.2)		23 (43.4)		81 (35.5)		186 (34.8)		350 (31.5)					
**Human epidermal growth factor receptor 2 status**															.89
	Positive	545 (28.3)		16 (30.2)		69 (30.3)		149 (27.9)		311 (28)					
	Negative	1383 (71.7)		37 (69.8)		159 (69.7)		386 (72.1)		801 (72)					
**Molecular subtype**															.43
	Luminal A	346 (17.9)		3 (5.7)		42 (18.4)		102 (19.1)		199 (17.9)					
	Luminal B	1059 (54.9)		32 (60.4)		125 (54.8)		280 (52.3)		622 (55.9)					
	HER-2 positive	194 (10.1)		6 (11.3)		20 (8.8)		54 (10.1)		114 (10.3)					
	Triple negative	329 (17.1)		12 (22.6)		41 (18)		99 (18.5)		177 (15.9)					
**Surgery**															<.001
	Breast conserving surgery	335 (17.4)		15 (28.3)		58 (25.4)		105 (19.6)		157 (14.1)					
	Mastectomy	1534 (79.5)		35 (66)		165 (72.4)		409 (76.5)		925 (83.2)					
	Unknown	59 (3.1)		3 (5.7)		5 (2.2)		21 (3.9)		30 (2.7)					
**Neoadjuvant chemotherapy**															<.001
	Yes	382 (19.8)		15 (28.3)		69 (30.3)		107 (20)		191 (17.2)					
	No	1546 (80.2)		38 (71.7)		159 (69.7)		428 (80)		921 (82.8)					
**Adjuvant chemotherapy**															.006
	Yes	1731 (89.8)		53 (100)		201 (88.2)		466 (87.1)		1011 (90.9)					
	No	197 (10.2)		0 (0)		27 (11.8)		69 (12.9)		101 (9.1)					
**Radiotherapy**															.02
	Yes	881 (45.7)		35 (66)		110 (48.2)		242 (45.2)		494 (44.4)					
	No	1047 (54.3)		18 (34)		118 (51.8)		293 (54.8)		618 (55.6)					
**Endocrine therapy**															.22
	Yes	1389 (72)		37 (69.8)		175 (76.8)		372 (69.5)		805 (72.4)					
	No	539 (28)		16 (30.2)		53 (23.2)		163 (30.5)		307 (27.6)					
**Targeted therapy**															.04
	Yes	340 (17.6)		11 (20.8)		50 (21.9)		106 (19.8)		173 (15.6)					
	No	1588 (82.4)		42 (79.2)		178 (78.1)		429 (80.2)		939 (84.4)					

### Trends in Annual Diagnosis by Age

The trends in the diagnosis of BC in the whole cohort and patients aged 18 to 25, 26 to 30, 31 to 35, and 36 to 40 years from 2008 to 2019 are presented in Table 2. The proportion of young BC patients significantly increased from 4.7% (n=92) in 2008 to 12.8% (n=247) in 2019. When stratified by age, annual diagnosis of patients aged 36 to 40 years significantly decreased from 59.4% (52/92) in 2008 to 44.6% (86/194) in 2018, while the diagnosis of patients aged 26 to 30 showed a significant upward trend, from 5.9% (6/92) in 2008 to 17.5% (34/194) in 2018. However, there were no significant tendencies in the diagnosis of patients aged 18 to 25 years, with 3% (3/92) in 2008 and 5.1% (10/194) in 2018; for patients aged 31 to 35 years, diagnosis was 31.7% (31/92) in 2008 and 32.8% (64/194) in 2018 (Table 2).

**Table 2:** Trends of annual diagnosis of patients in different age groups (18-25, 26-30, 31-35, and 36-40 years).

Year	Age group (years), n (%)			
	18-25	26-30	31-35	36-40
2008 (n=92)	3 (3)	6 (5.9)	31 (31.7)	52 (59.4)
2009 (n=140)	5 (4.2)	11 (7.7)	36 (25.9)	88 (62.2)
2010 (n=120)	1 (0.8)	15 (12.5)	26 (21.7)	78 (65)
2011 (n=152)	3 (1.9)	10 (6.5)	32 (20.8)	107 (70.8)
2012 (n=160)	4 (2.5)	13 (8)	41 (25.9)	102 (63.6)
2013 (n=146)	3 (2.7)	9 (6.1)	30 (20.3)	104 (70.9)
2014 (n=144)	3 (2.1)	17 (11.8)	33 (22.9)	91 (63.2)
2015 (n=176)	3 (1.7)	26 (15.3)	50 (28.2)	97 (54.8)
2016 (n=149)	7 (4.7)	23 (15.4)	40 (26.8)	79 (53)
2017 (n=208)	5 (2.4)	40 (19.2)	68 (32.7)	95 (45.7)
2018 (n=194)	10 (5.1)	34 (17.5)	64 (32.8)	86 (44.6)
2019 (n=247)	6 (2.4)	24 (9.6)	84 (33.7)	133 (54.2)

### Survival and Prognostic Analysis by Age

**Figure 2:**
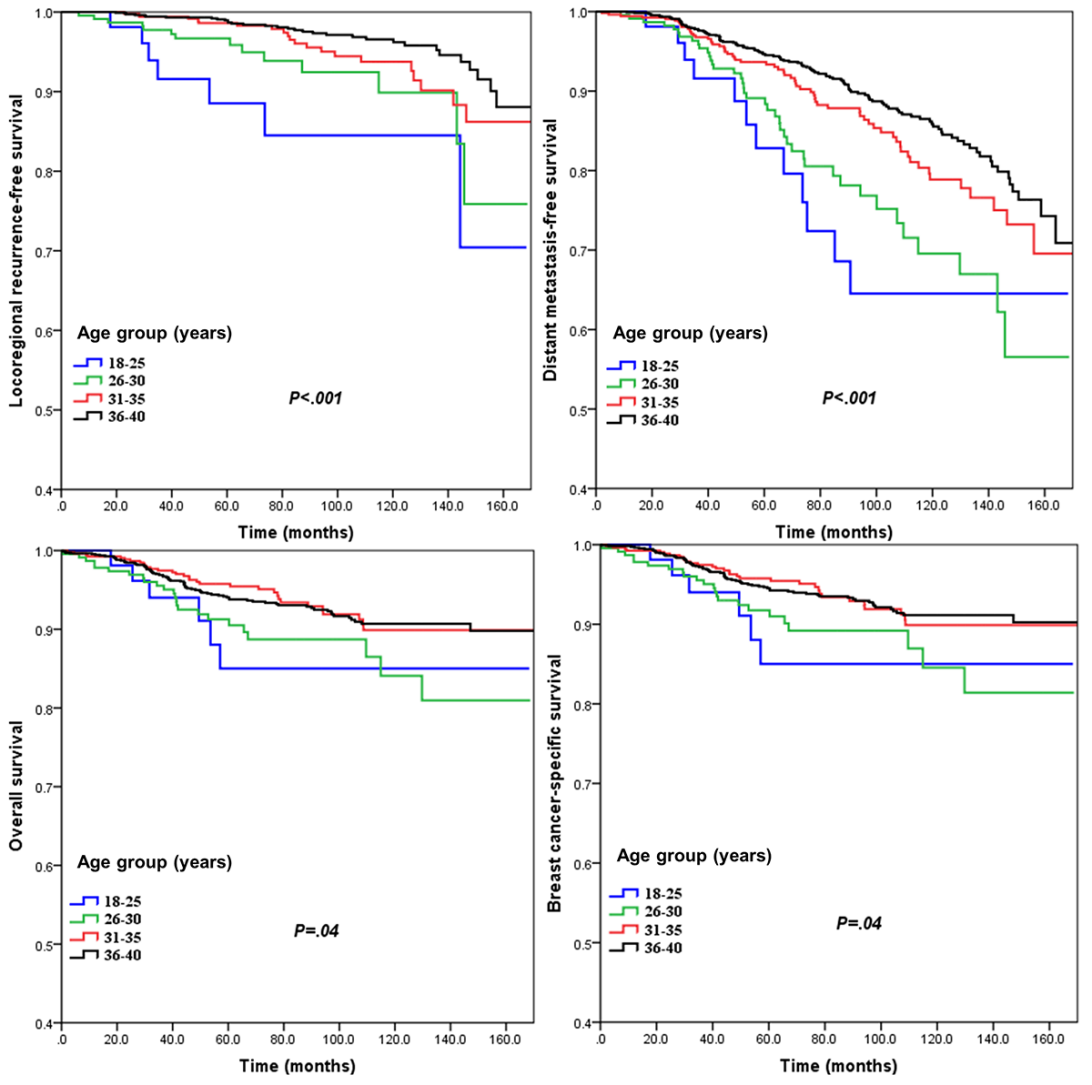
Survival curves of locoregional recurrence–free survival, distant metastasis–free survival, breast cancer–specific survival, and overall survival for young patients of different ages.

With a median follow-up time of 75.7 months (range 1.1-173 months), 75 (3.9%) recurrences, 236 (12.2%) cases of distant metastasis, 132 (6.8%) BC-related deaths, and 138 (7.2%) other deaths occurred among the 1928 participants. In the entire group, 5-year LRFS, DMFS, BCSS, and OS were 98.3%, 93.4%, 94.4%, and 94%, respectively. There were better survival outcomes with age in BC patients aged ≤40 years. Patients aged 18 to 25 years had significantly poorer 5-year LRFS (ages 18-25, 26-30, 31-35, and 36-40 years: 88.5%, 96.7%, 98.6%, and 98.9%, respectively; *P*<.001; Figure 2A), 5-year DMFS (ages 18-25, 26-30, 31-35, and 36-40 years: 82.8%, 89.1%, 93.7%, and 94.6%, respectively; *P*<.001; Figure 2B), 5-year BCSS (ages 18-25, 26-30, 31-35, and 36-40 years: 85%, 91.8%, 95.8%, and 94.5%, respectively; *P*=.04; Figure 2C), and 5-year OS (age 18-25, 26-30, 31-35, and 36-40 years: 85%, 91.3%, 95.8%, and 94%, respectively; *P*=.04; Figure 2D) than those aged 31-35, 26-30, and 36-40 years. Cox multivariate regression model showed that age was a significant predictor for LRFS (hazard ratio [HR] 0.645, 95% CI 0.500-0.831; *P*<.001) and DMFS (HR 0.743, 95% CI 0.641-0.861; *P*<.001), while it was not a predictor for BCSS (HR 0.889, 95% CI 0.729-1.805; *P*=.25) or OS (HR 0.867, 95% CI 0.709-1.061; *P*=.17; Table 3).

**Table 3:** Cox multivariate analysis of locoregional recurrence–free survival, distant metastasis–free survival, breast cancer–specific survival, and overall survival in all patients.

Outcome and age group (years)		Hazard ratio (95% CI)	*P* value	
**Locoregional recurrence–free survival**				
	18-25	1		
	26-30	0.551 (0.193-1.571)	.27	
	31-35	0.280 (0.105-0.752)	.01	
	36-40	0.228 (0.088-0.590)	.002	
**Distant metastasis–free survival**				
	18-25	1		
	26-30	0.967 (0.489-1.910)	.92	
	31-35	0.539 (0.280-1.035)	.06	
	36-40	0.448 (0.238-0.845)	.01	
**Breast cancer–specific survival**				
	18-25	1		
	26-30	0.959 (0.378-2.432)	.93	
	31-35	0.518 (0.210-1.227)	.15	
	36-40	0.596 (0.252-1.410)	.24	
**Overall survival**				
	18-25	1		
	26-30	0.997 (0.395-2.515)	.99	
	31-35	0.517 (0.210-1.272)	.15	
	36-40	0.644 (0.273-1.518)	.32	

### Annualized Hazard Curve of Recurrence, Metastasis, and BC Death

**Figure 3:**
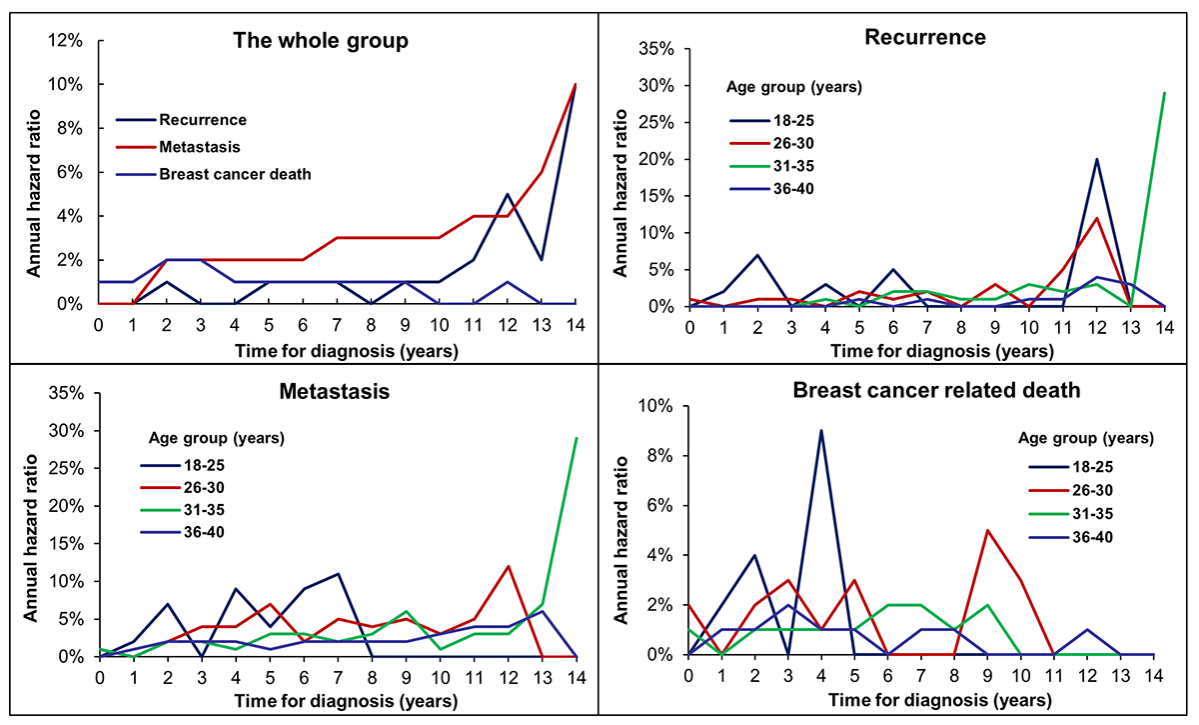
Annualized hazard curves for recurrence, metastasis, and breast cancer–related mortality for the whole group and distinct age strata of the patients.

We also explored the annualized hazard trends of recurrence, metastasis, and BC mortality in this population (Figure 3). The hazard curves for recurrence and metastasis in the whole cohort continuously increased over time and did not reach a peak within the follow-up of 14 years. The BC mortality risk curve peaked at 2 to 3 years (at 2%), maintained a steady lower level after 3 years, then changed to a slowly decreasing plateau (Figure 3). After conducting a stratified analysis by age, we found that patients aged 18 to 25 years had peak BC mortality at 2 and 4 years and still had peak metastasis risk before the first 8 years. Patients aged 26 to 30, 31 to 35, and 36 to 40 years all showed a slow upward trend of recurrence and metastasis risk during follow-up. In addition, patients aged 26 to 30 years had high risk of BC mortality during years 0 to 6 and 8 to 11, while patients aged 31 to 35 years had high risk before the first 10 years. The hazard function for BC mortality in patients aged 36 to 40 years maintained a high plateau before the first 8 years then changed to a low death rate (Figure 3).

## Discussion

### Principal Findings

We provide updated data on the clinicopathological characteristics, survival outcomes, and hazard function of different age strata of Chinese BC patients aged younger than 40 years. The main finding was that different age strata of young BC patients had different rates of survival: the younger the patient, the worse the prognosis. In addition, we found the risk of recurrence and metastasis continuously increased over time, and the failure patterns of different age strata were significantly different. Our result demonstrating different survival outcomes and failure patterns in different age strata could be used to tailor personalized management of young BC patients.

Previous studies have demonstrated that young BC patients have worse prognoses than older patients; 5-year BCSS in patients aged ≤35 years was approximately 80%, while it was 90% in patients ≥60 years [7,20]. The reasons for this biological difference between young and old patients have not been well elaborated. Several possible reasons might explain the phenomenon. First, younger patients are more likely to develop tumors with aggressive characteristics, such as higher Ki-67 and a triple-negative subtype [7-12]. Second, younger patients have higher expression of RANK-ligand, c-kit, mammary stem cell, and BRCA1 mutation signatures and the deregulation of PI3K and Myc pathways, which are associated with a poor prognosis [21-23]. Third, young BC patients have increased risk of psychosocial stress, and their treatment strategies are more likely to be affected by considerations of sexual function, fertility, beauty, body image, and their careers [24,25]. In this study, the 5-year BCSS and OS in young patients were 94.3% and 93.9%, respectively. These survival rates were higher than those of older Chinese patients in a previous study; the reason might be that more patients had early-stage tumors (6279/7553, 83.1%), as well as nodal-stage (6821/7553, 90.3%) and luminal-subtype (5937/7553, 78.6%) tumors in this study [26]. In addition, survival outcomes among younger patients in our study were also higher than those in younger women in the United States, which might be attributable to racial, dietary, climate, and living differences leading to Asian BC patients having better survival than White patients [7,26,27].

It has not been fully delineated whether survival and prognoses are the same in different age strata among young women. A study conducted by Fredholm et al [28] included 1120 women with stage I to III cancer; their results showed that patients aged <30 years had significantly poorer 5-year BCSS than those aged 35 to 40 years (80% vs 86%; *P*<.001). However, the opposite result was found in another population-based report in Italy: there was no survival difference among patients aged <25, 25 to 29, and 30 to 34 years in locoregional relapse (*P*=.87), distant metastasis (*P*=.40), BCSS (*P*=.58), and OS (*P*=.99) [27]. In our study, there were significant differences in LRFS (*P*<.001), DMFS (*P*<.001), BCSS (*P*=.035), and OS (*P*=.037) in younger patients by age, and patients aged <25 years old had the worst survival rate. The reasons for the difference might be that the sample size in the previous study was small (n=497) and that the included patients were White; these are important differences from our study [27]. Therefore, it is important to establish policies for better managing and improving the survival of very young Chinese BC patients.

There is limited evidence available evaluating the risk of relapse and death in young BC patients. A recent analysis from the International Breast Cancer Study Group clinical trials included 4105 BC patients and explored the patterns of late BC recurrence. The authors demonstrated that the annualized hazard of recurrence peaked at 2 years and then decreased slowly; however, the study did not stratify the analysis for young BC patients [29]. Another study by de la Rochefordiere and his colleagues [30] assessed failure patterns in young age groups. They included 3371 women aged ≤55 years and divided them into 3 groups (≤33 years, 34-40 years, and ≥40 years). The results showed that the annual hazard rate of relapse peaked at 2 years and patients aged ≤33 years had a higher relapse risk than those aged 34 to 40 years for about 5 years, but a lower risk after 5 years [30]. A similar result was found in our study: BC death risk peaked at 2 and 3 years after diagnosis; however, totally different trends were observed for the annual hazard of recurrence and metastasis, which both showed a slow upward trend in our study that did not reach a peak during follow-up, possibly attributable to differences in race and sample size between the studies (1950 patients in this study vs 456 patients in the past study) [29]. The reasons for the different failure patterns might include differences in the expression of RANK-ligand, c-kit, mammary stem cell, and BRCA1 mutation signatures, as well as in the PI3K and Myc pathways [21-23]. Therefore, it is essential to further explore the optimal management of young BC patients to reduce the risk of recurrence and metastasis.

### Limitations

Our study has several limitations that might affect the results. First, the patient data were extracted from a database that was built beforehand; selection bias in retrospective studies is inevitable. Second, our data were collected at a single center, and the result thus cannot represent all patients in China. Third, this study only included hospitalized patients, which probably does not represent all patients with cancer (ie, there may be patients that have cancer but are not hospitalized). Finally, the sample size in our study was relatively small, especially for patients aged 18 to 25 years. Therefore, a multicenter prospective study with a larger sample size should be conducted to further explore the characteristics of young BC patients and their management. Despite the limitations of our study, we have expanded the understanding of young BC in different races and ethnicities.

### Conclusions

Our study demonstrates that the annual diagnosis of young BC increased in Chinese patients. The diagnostic rate of patients aged 36 to 40 years decreased, while it remained stable in those aged 18 to 25 and 31 to 35 years. Young BC patients of different age groups did not differ in survival outcomes, but the younger the patient, the worse were the 5-year LRFS, DMFS, BCSS, and OS. In addition, there were significantly different failure patterns in different age strata among the young BC patients, especially for BC mortality. Our results demonstrating different survival outcomes and failure patterns in different age strata may allow tailored, personalized management for young BC patients.

## Abbreviations

BC: breast cancer
BCSS: breast cancer–specific survival
DMFS: distant metastasis–free survival
ER: estrogen receptor
HER-2: human epidermal growth factor receptor 2
HR: hormone receptor
LRFS: locoregional recurrence–free survival
OS: overall survival
PR: progesterone receptor
TIS: tumor in situ

## Data Availability

The data sets generated during and/or analyzed during this study are available from the corresponding author on reasonable request.
